# Short-Term Metabolic and Inflammatory Effects of Upadacitinib in Biologic-Refractory Spondyloarthritis: Real-World Evidence on Lipid Paradox and Safety

**DOI:** 10.3390/pharmaceutics18020272

**Published:** 2026-02-22

**Authors:** Zeynel Abidin Akar, Dilan Yıldırım, Ömer Karakoyun, Mehmet Çağlayan

**Affiliations:** 1 Division of Rheumatology, Department of Physical Therapy and Rehabilitation, Faculty of Medicine, Dicle University, 21280 Diyarbakır, Türkiye; 2 Department of Physical Therapy and Rehabilitation, Faculty of Medicine, Dicle University, 21280 Diyarbakır, Türkiye; dilanyildirim484@gmail.com; 3 Department of Dermatology and Venereology, Faculty of Medicine, Dicle University, 21280 Diyarbakır, Türkiye; omerkarakoyun@gmail.com

**Keywords:** upadacitinib, spondyloarthritis, JAK inhibitors, lipid profile, lipid paradox, real-world study

## Abstract

**Background:** Upadacitinib (UPA), a selective Janus kinase 1 (JAK1) inhibitor, is an established therapeutic option for spondyloarthritis (SpA). Although its clinical efficacy has been demonstrated in randomized trials, real-world evidence regarding its early metabolic effects—particularly in the context of the inflammatory “lipid paradox”—remains limited. This study aimed to evaluate the short-term impact of UPA on inflammatory, hematologic, and metabolic parameters in a biologic-refractory SpA cohort. **Methods:** This retrospective cohort study included 61 patients (51 with ankylosing spondylitis and 10 with psoriatic arthritis) who had an inadequate response to tumor necrosis factor inhibitors (TNFi-IR). The study evaluated the short-term effects of UPA treatment on disease activity, inflammatory markers, and lipid-related atherogenic risk, as assessed using the LDL/HDL ratio, over a three-month period. Demographic characteristics, disease activity (BASDAI), inflammatory markers (CRP, ESR), safety parameters (creatine kinase [CK], ALT, AST), and lipid profiles were assessed at baseline, Month 1, and Month 3. **Results:** The mean age was 42.6 ± 10.8 years. By Month 3, UPA treatment resulted in significant reductions in BASDAI (5.8 ± 1.4 to 3.6 ± 1.2, *p* < 0.001), CRP (11.4 ± 10.2 to 6.9 ± 5.8 mg/L), and ESR (*p* < 0.01). Hemoglobin and albumin levels increased significantly (*p* < 0.05), while liver enzymes remained stable. CK levels demonstrated a modest but statistically significant increase without exceeding three times the upper limit of normal and without clinical evidence of myopathy. Total cholesterol, LDL-C, and HDL-C increased significantly (*p* ≤ 0.003); however, triglycerides and the LDL/HDL ratio remained unchanged (*p* > 0.05). No significant differences in inflammatory or metabolic responses were observed between ankylosing spondylitis and psoriatic arthritis subgroups (*p* > 0.05). **Conclusions:** In biologic-refractory SpA patients, upadacitinib provides rapid anti-inflammatory and clinical benefits. Although quantitative increases in lipid subfractions were observed, the stability of the LDL/HDL ratio suggests a balanced metabolic recalibration consistent with inflammation control rather than an immediate pro-atherogenic shift. These findings highlight the importance of early lipid monitoring and individualized cardiovascular risk assessment while maintaining the therapeutic advantages of JAK1 inhibition in complex SpA populations.

## 1. Introduction

Spondyloarthritis (SpA) comprises a heterogeneous group of chronic inflammatory rheumatic diseases, including axial spondyloarthritis (axSpA) and psoriatic arthritis (PsA). These conditions are characterized by progressive structural damage, chronic pain, and functional impairment affecting the axial skeleton, peripheral joints, and entheses [1]. Beyond musculoskeletal involvement, persistent systemic inflammation in SpA is associated with an increased risk of comorbidities, particularly accelerated atherosclerosis and cardiovascular disease (CVD) [2]. Although biologic therapies—such as tumor necrosis factor inhibitors (TNFi) and interleukin-17 inhibitors (IL-17i)—have substantially improved disease outcomes, a considerable proportion of patients remain refractory or experience inadequate response, highlighting the need for alternative targeted treatment strategies in difficult-to-treat SpA [3].

The management of complex SpA phenotypes increasingly requires an integrated and multidisciplinary approach, addressing not only articular manifestations but also systemic comorbidities and metabolic health. Chronic inflammation in SpA is closely linked to alterations in lipid metabolism and nutritional status. Paradoxically, active inflammatory states are often associated with reduced circulating lipid levels despite increased cardiovascular risk—a phenomenon known as the “lipid paradox.” Effective anti-inflammatory therapy may reverse this pattern, leading to quantitative increases in lipid fractions that reflect metabolic normalization rather than true pro-atherogenic worsening [4]. Emerging composite indices integrating inflammatory and nutritional markers—such as the Prognostic Inflammatory and Nutritional Index (PINI)—have been proposed in other chronic inflammatory conditions to better characterize systemic burden and metabolic risk, and may represent promising tools for future investigation in SpA populations [5].

The Janus kinase–signal transducer and activator of transcription pathway plays a central role in mediating the signaling of multiple pro-inflammatory cytokines implicated in SpA pathogenesis, including interleukin-6 and interferon-γ [6]. Upadacitinib, a selective Janus kinase 1 (JAK1) inhibitor, has demonstrated significant efficacy in both axSpA and PsA across phase 3 clinical trials, including biologic-naïve and TNFi-refractory populations [7]. However, JAK inhibitors are known to induce quantitative changes in serum lipid subfractions, and mild elevations in creatine kinase (CK) have also been reported in clinical studies [8]. Most available evidence derives from randomized controlled trials with strict inclusion criteria, whereas real-world data capturing early metabolic adaptations and safety signals in biologic-refractory SpA populations remain limited.

In rheumatoid arthritis populations, JAK inhibitor therapy has consistently been associated with increases in total cholesterol (TC), low-density lipoprotein cholesterol (LDL-C), and high-density lipoprotein cholesterol (HDL-C), while the long-term cardiovascular implications of these changes continue to be debated [9,10]. Data specifically addressing early lipid dynamics in SpA populations are comparatively scarce. Given the intrinsically elevated cardiovascular risk associated with chronic systemic inflammation in SpA, the early characterization of lipid changes following JAK1 inhibition is clinically important for timely cardiovascular risk stratification and monitoring [11,12,13].

Therefore, the aim of this study was to evaluate the short-term effects of upadacitinib on disease activity, inflammatory markers, hematologic indices, and lipid profiles in biologic-experienced patients across the spondyloarthritis spectrum in a real-world clinical setting. The null hypothesis was that upadacitinib treatment would not result in significant modifications in inflammatory parameters or lipid-related atherogenic risk, as assessed using the LDL/HDL ratio, within three months of therapy initiation.

## 2. Materials and Methods

### 2.1. Study Design and Patient Population

This retrospective cohort study was conducted at the Rheumatology Outpatient Clinic of Dicle University Faculty of Medicine. Medical records of patients within the Spondyloarthritis (SpA) spectrum—including ankylosing spondylitis (AS) and psoriatic arthritis (PsA)—who initiated upadacitinib therapy (15 mg once daily) were reviewed. All patients had demonstrated inadequate response or intolerance to at least one tumor necrosis factor inhibitor (TNFi), including etanercept, adalimumab, infliximab, certolizumab, or golimumab (TNFi-IR).

Eligible patients were required to have a confirmed diagnosis according to either the Modified New York Criteria [14] or the ASAS classification criteria for axial SpA [15], or the CASPAR criteria for PsA [16]. All participants received upadacitinib for clinical indications determined by the treating rheumatologist. Patients with a history of familial hyperlipidemia, those receiving lipid-lowering therapy at baseline, or those with incomplete clinical or laboratory data were excluded to ensure the validity of metabolic and lipid analyses.

**Figure 1:**
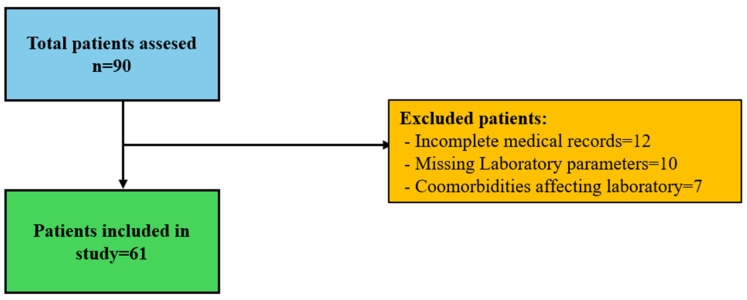
Patient Selection Flowchart. This flowchart illustrates the inclusion and exclusion process for SpA patients who initiated upadacitinib therapy. Inclusion criteria: inadequate response or intolerance to at least one TNF inhibitor, confirmed AS and PsA diagnosis according to the Modified New York or ASAS and CASPAR criteria. Exclusion criteria: history of familial hyperlipidemia, use of lipid-lowering therapy at baseline, or incomplete clinical/laboratory data.

The study protocol was approved by the Dicle University Faculty of Medicine Ethics Committee (Approval No: 6; Date: 24 December 2025) and conducted in accordance with the Declaration of Helsinki. The requirement for informed consent was waived due to the retrospective nature of the study. The patient selection process, reflecting the transition from the initial cohort to the final study population, is summarized in Figure 1.

### 2.2. Clinical and Laboratory Assessments

Baseline characteristics, disease duration, and concomitant medications—including NSAIDs, conventional synthetic DMARDs (csDMARDs), and low-dose oral corticosteroids (≤10 mg/day)—were extracted from electronic medical records. Disease activity was assessed using the Bath Ankylosing Spondylitis Disease Activity Index (BASDAI) at baseline, Month 1, and Month 3. Laboratory assessments were performed on fasting venous blood samples (≥8 h fasting) and included inflammatory markers (CRP, ESR), hematologic and nutritional indices (WBC, hemoglobin, platelets, serum albumin), metabolic and safety parameters (ALT, AST, fasting glucose, creatine kinase [CK]), and a detailed lipid profile (total cholesterol, LDL-C, HDL-C, triglycerides). Secondary indices such as non-HDL cholesterol and the LDL/HDL ratio were also calculated to evaluate the atherogenic profile.

### 2.3. Statistical Analysis

Statistical analyses were conducted using SPSS version 27.0 (IBM Corp., Armonk, NY, USA). The normality of continuous variables was assessed using the Kolmogorov–Smirnov test and visually confirmed with Q–Q plots. Longitudinal changes from baseline to Month 1 and Month 3 were analyzed using repeated-measures ANOVA or the non-parametric Friedman test, depending on data distribution. Post hoc pairwise comparisons were adjusted using the Bonferroni correction to control for Type I error across multiple time points.

To examine the consistency of findings across the spondyloarthritis spectrum, subgroup analyses were performed to compare clinical and metabolic changes between AS and PsA patients using independent-samples *t*-tests or Mann–Whitney U tests, as appropriate. Statistical significance was defined as *p* < 0.05.

A post hoc power analysis based on the primary outcome (BASDAI change) using G*Power v3.1.9.7, assuming an effect size of 0.5 and α = 0.05, yielded a statistical power greater than 90%, confirming the study was sufficiently powered to detect clinically meaningful differences in this cohort of 61 patients.

## 3. Results

The study cohort comprised 61 patients with a mean age of 42.6 ± 10.8 years. The majority were diagnosed with ankylosing spondylitis (AS; n = 51, 83.6%), while 10 patients (16.4%) had psoriatic arthritis (PsA). All participants were biologic-refractory, having demonstrated inadequate response or intolerance to at least one TNF inhibitor (TNFi-IR). Notably, 39.3% had failed two or more TNFi agents, and 22.9% had prior exposure to an IL-17 inhibitor (secukinumab), reflecting a difficult-to-treat population with high baseline disease activity (mean BASDAI: 5.8 ± 1.4). No significant baseline differences were observed between AS and PsA subgroups in demographic, inflammatory, or metabolic parameters (*p* > 0.05). Baseline characteristics are summarized in Table 1.

**Figure 2:**
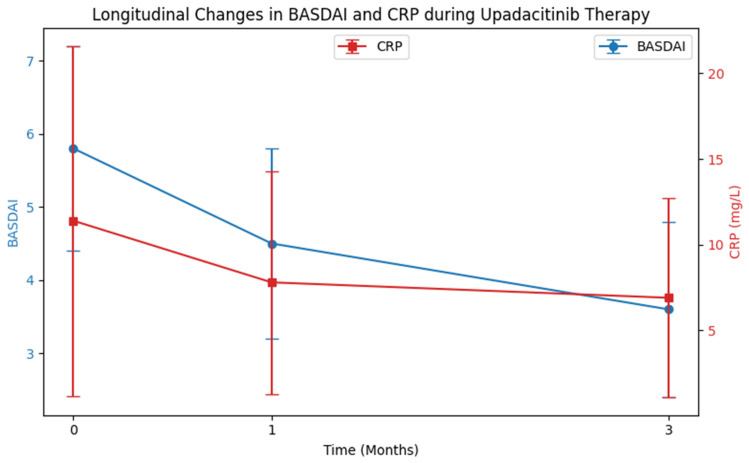
Short-term effects of upadacitinib on disease activity and inflammation. Line graphs depict the Bath Ankylosing Spondylitis Disease Activity Index (BASDAI) scores (left axis) and C-reactive protein (CRP, mg/L; right axis) at baseline, Month 1, and Month 3. Both parameters show a marked decrease, indicating that upadacitinib exerts rapid clinical and anti-inflammatory effects within the first month of treatment.

Longitudinal analysis demonstrated a rapid and significant clinical response to upadacitinib (Table 2). BASDAI scores decreased from 5.8 ± 1.4 at baseline to 3.6 ± 1.2 by Month 3 (*p* < 0.001). Parallel reductions were observed in inflammatory markers, with CRP declining from 11.4 ± 10.2 mg/L to 6.9 ± 5.8 mg/L (*p* < 0.001) and ESR decreasing significantly over time (*p* = 0.002). These improvements were already evident at Month 1 and were sustained through Month 3 (Figure 2).

Laboratory monitoring indicated a favorable short-term safety profile (Table 3). Hemoglobin increased from 13.6 ± 1.4 to 14.1 ± 1.3 g/dL (*p* = 0.032), while albumin levels rose from 4.1 ± 0.4 to 4.4 ± 0.3 g/dL (*p* = 0.005). Platelet counts showed a modest but statistically significant reduction (*p* = 0.028), whereas leukocyte and lymphocyte counts remained stable.

Creatine kinase (CK) levels increased modestly from 112.4 ± 54.8 to 144.2 ± 68.5 U/L (*p* = 0.012), without exceeding three times the upper limit of normal or requiring treatment discontinuation. Liver enzymes (ALT, AST) and fasting glucose levels did not change significantly during follow-up (*p* > 0.05).

Quantitative increases were observed in several lipid subfractions during follow-up (Table 4 and Table 5). By Month 3, total cholesterol increased from 199.4 ± 41.2 to 215.6 ± 35.4 mg/dL (*p* = 0.001), and LDL-C rose from 118.7 ± 33.6 to 136.5 ± 29.8 mg/dL (*p* = 0.003), corresponding to a mean increase of +17.8 ± 8.4 mg/dL. HDL-C also increased significantly (+8.5 ± 4.1 mg/dL; *p* < 0.001). These changes were already detectable at Month 1.

**Figure 3:**
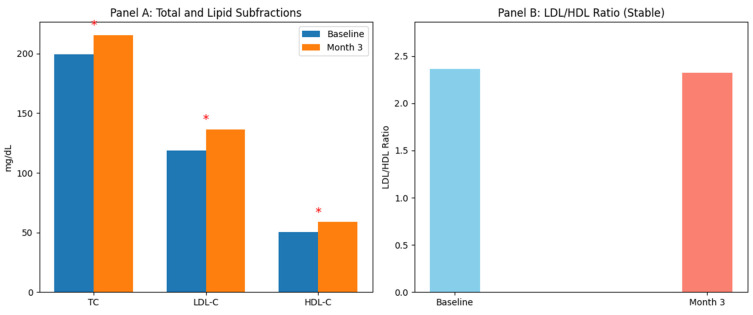
Short-term effects of upadacitinib on lipid profile. (**Panel A**) Total cholesterol (TC), low-density lipoprotein cholesterol (LDL-C), and high-density lipoprotein cholesterol (HDL-C) levels at baseline and Month 3. Asterisks (*) indicate *p* < 0.05, representing statistically significant increases. (**Panel B**) LDL/HDL ratio at baseline and Month 3. Thin bars with different colors were used (baseline: blue, Month 3: salmon). The ratio remained stable, demonstrating that, despite increases in lipid subfractions, the atherogenic profile is preserved.

In contrast, triglyceride levels did not significantly change at either time point (*p* > 0.05). Importantly, the LDL/HDL ratio remained stable (baseline: 2.36 ± 0.8 vs. Month 3: 2.32 ± 0.6; *p* = 0.845), indicating a preservation of the atherogenic index despite quantitative lipid elevations. Non-HDL cholesterol showed a modest but statistically significant increase (*p* = 0.008). Figure 3 illustrates these short-term lipid dynamics.

The subgroup analysis of changes from baseline to Month 3 (Table 5) demonstrated consistent responses across the SpA spectrum. Both AS and PsA patients experienced significant reductions in disease activity (ΔBASDAI: −2.2 ± 1.1 vs. −2.4 ± 1.3; *p* = 0.612) and CRP (−4.6 ± 3.2 mg/L vs. −4.1 ± 3.8 mg/L; *p* = 0.658).

Similarly, LDL-C and HDL-C increased in both groups without significant between-group differences (*p* = 0.424 and *p* = 0.218, respectively). The LDL/HDL ratio remained stable in both AS (−0.04 ± 0.14) and PsA (−0.06 ± 0.18) subgroups (*p* = 0.715). Albumin levels increased comparably (*p* = 0.384). These findings indicate homogeneous clinical and metabolic responses to upadacitinib across diagnostic subtypes within the spondyloarthritis spectrum.

## 4. Discussion

In this real-world cohort of biologic-experienced patients across the Spondyloarthritis (SpA) spectrum, including AS and PsA, upadacitinib treatment demonstrated a rapid and robust clinical impact, with significant reductions in disease activity and systemic inflammatory markers within three months. BASDAI scores, CRP levels, and ESR declined markedly, consistent with pivotal clinical trials such as SELECT-AXIS 1 and 2, confirming the efficacy of selective JAK1 inhibition even in a refractory population with prior TNF inhibitor exposure [17,18]. Laboratory monitoring showed stable hematologic parameters and liver enzymes, supporting the short-term safety profile of treatment. Notably, significant increases in albumin and hemoglobin levels were observed (*p* < 0.05); as suggested by Cordos et al., such shifts in nutritional and inflammatory markers are important indicators of systemic recovery and may reflect improved metabolic homeostasis in chronic inflammatory diseases [5].

Quantitative increases were observed in total cholesterol, LDL-C, and HDL-C levels, whereas the LDL/HDL ratio and triglycerides remained stable, indicating preservation of the atherogenic index. This pattern aligns with the concept of the “lipid paradox,” whereby active systemic inflammation suppresses circulating lipid concentrations despite increased cardiovascular risk [19,20]. The effective suppression of inflammatory cytokines through JAK1 inhibition appears to reverse this phenomenon, resulting in a parallel rise in both LDL-C and HDL-C without qualitative pro-atherogenic imbalance [21]. The stability of non-HDL cholesterol and triglyceride levels further supports the interpretation of lipid recalibration rather than the induction of a de novo atherogenic state [22].

These findings are consistent with meta-analytic evidence demonstrating similar lipid modifications across JAK inhibitor therapies [23]. Comparable lipid elevations have been reported with Tofacitinib and Filgotinib, where parallel increases in LDL-C and HDL-C occur without significant alteration of the LDL/HDL ratio [24]. The preferential JAK1 selectivity of Upadacitinib may theoretically allow the targeted modulation of IL-6–driven inflammatory pathways central to SpA pathogenesis while maintaining a predictable metabolic profile; however, definitive comparative cardiovascular safety differences among JAK inhibitors remain to be established in head-to-head real-world studies.

The rapid and significant reductions in BASDAI scores and inflammatory markers observed in our cohort mirror outcomes from SELECT-AXIS trials [17,18], supporting the reproducibility of JAK1 inhibition efficacy in routine clinical practice. Subgroup analysis demonstrated comparable clinical and metabolic responses between AS and PsA patients (*p* > 0.05), reinforcing the therapeutic versatility of JAK1 inhibition across the broader SpA spectrum.

The observed increases in albumin levels (*p* = 0.005) further substantiate the concept of systemic recovery. Albumin serves as a sensitive marker of the interplay between inflammation and nutritional status, and its rise may indicate the restoration of metabolic balance following effective cytokine suppression [5]. Together with lipid normalization, these findings suggest a broader anti-inflammatory and metabolic recalibration effect.

The clinical efficacy of upadacitinib is attributable to the selective inhibition of JAK1-mediated signaling pathways involving IL-6, IL-12, and IL-23, which are central to SpA immunopathogenesis [25,26]. By disrupting these cytokine cascades, upadacitinib achieves a rapid suppression of systemic inflammation, reflected by significant improvements in both objective (CRP, ESR) and patient-reported (BASDAI) outcomes [27].

From a practical clinical perspective, early lipid elevations during upadacitinib therapy should not automatically prompt treatment discontinuation. Instead, they should be interpreted within the broader context of inflammation control and global cardiovascular (CV) risk assessment [28]. Baseline lipid profiling, followed by reassessment within the first 8–12 weeks, is advisable [29]. Lipid-lowering therapy, including statins, should be initiated according to established CV prevention guidelines and individualized risk stratification rather than isolated lipid changes alone [30]. This strategy enables clinicians to preserve the substantial anti-inflammatory benefits of JAK1 inhibition while proactively mitigating potential metabolic risks.

Given the intrinsically elevated CV burden in SpA [31], careful longitudinal monitoring remains essential. Although short-term LDL/HDL ratio stability suggests a neutral early atherogenic profile, long-term prospective studies are required to determine whether sustained lipid elevations influence major adverse cardiovascular event (MACE) risk [32]. The present study intentionally focused on early (1- and 3-month) outcomes, as lipid modifications associated with JAK inhibition are known to occur rapidly after treatment initiation, making early evaluation clinically relevant for timely cardiovascular risk management.

Regarding muscle safety, our analysis demonstrated a statistically significant but clinically modest increase in creatine kinase (CK) levels. CK elevations have previously been described with JAK inhibitors and are typically asymptomatic and reversible. Although the precise mechanism remains unclear, it may relate to altered cytokine-mediated muscle signaling or subclinical myocyte turnover [33,34]. In our cohort, CK values did not exceed three times the upper limit of normal and did not necessitate treatment discontinuation, supporting the short-term muscular safety of upadacitinib in real-world practice.

Several strengths enhance the clinical relevance of our findings. The study provides real-world evidence in a fully TNFi-inadequate responder population, a difficult-to-treat group often underrepresented in randomized trials. The simultaneous evaluation of disease activity indices, inflammatory biomarkers, CK levels, and detailed lipid subfractions enabled a comprehensive assessment of efficacy and metabolic safety. The inclusion of consecutive patients from a tertiary referral center minimized selection bias and reflected routine rheumatologic care. Early longitudinal assessment captured the kinetics of both inflammatory suppression and lipid recalibration, offering practical guidance for early monitoring strategies.

Nevertheless, important limitations should be acknowledged. The retrospective single-center design limits generalizability and precludes causal inference. No a priori sample size calculation was performed due to the retrospective design; therefore, the findings should be interpreted as exploratory and hypothesis-generating. The relatively modest sample size, particularly within subgroup analyses, may reduce sensitivity to detect rare adverse events. The three-month follow-up restricts conclusions regarding the long-term durability of responses and cardiovascular outcomes. Furthermore, the absence of a parallel control group necessitates the cautious interpretation of treatment-attributable effects. Finally, a reliance on electronic medical records may introduce information bias.

Future research should prioritize long-term, prospective multicenter studies in biologic-experienced SpA populations to confirm the sustainability of clinical responses and clarify cardiovascular implications of persistent lipid modifications. Extended follow-up is essential to determine whether early lipid recalibration translates into meaningful long-term CV outcomes. Moreover, integrating composite inflammatory–nutritional indices such as the Prognostic Inflammatory and Nutritional Index (PINI), as highlighted by Cordos et al. [5], may provide deeper insight into systemic recovery following JAK inhibition. Comparative real-world investigations evaluating JAK inhibitors and alternative biologic strategies may further refine therapeutic positioning and sequencing in refractory SpA.

## 5. Conclusions

This real-world study demonstrates that upadacitinib provides rapid and substantial clinical improvement with potent anti-inflammatory effects in biologic-refractory patients across the spondyloarthritis spectrum. Within the first three months of therapy, total cholesterol, LDL-C, and HDL-C increased quantitatively; however, the LDL/HDL ratio remained stable. Alongside significant increases in serum albumin, these findings suggest a metabolic normalization consistent with the reversal of the inflammatory lipid paradox rather than an immediate pro-atherogenic shift. Subgroup analyses confirmed that these clinical and metabolic benefits are consistent across both ankylosing spondylitis and psoriatic arthritis phenotypes.

Overall, upadacitinib demonstrates a favorable short-term safety and efficacy profile in a difficult-to-treat, biologic-experienced population. Given the chronic course of spondyloarthritis and its associated cardiovascular risk, these results underscore the importance of proactive metabolic monitoring. A multidisciplinary management strategy that combines rapid disease control with vigilant cardiovascular risk assessment can optimize holistic care for patients with complex spondyloarthritis.

## Figures and Tables

**Table 1:** Demographic and Baseline Clinical Characteristics of Participants (n = 61).

Variable	Value (n = 61)
Age (years)	42.6 ± 10.8
Sex, n (%)	
Female	27 (44.3)
Male	34 (55.7)
Disease duration (years)	8 [4–13]
Diagnosis, n (%)	
Ankylosing Spondylitis (AS)	51 (83.6)
Psoriatic Arthritis (PsA)/Other	10 (16.4)
Baseline disease activity, Mean ± SD	
BASDAI score	5.8 ± 1.4
CRP (mg/L)	11.4 ± 10.2
ESR (mm/h)	14.6 ± 11.5
History of biologic therapy, n (%)	
Biologic-experienced (TNFi-IR)	61 (100)
Used 1 TNF inhibitor	37 (60.7)
Used 2 TNF inhibitors	18 (29.5)
Used ≥ 3 TNF inhibitors	6 (9.8)
Previous Secukinumab (IL-17i) use	14 (22.9)
Concomitant treatments, n (%)	
Conventional DMARDs (MTX, SSZ, LEF)	11 (18.0)
NSAID use	18 (29.5)
Low-dose glucocorticoids	6 (9.8)

Data are presented as mean ± standard deviation or median [interquartile range] for continuous variables and as number (percentage) for categorical variables. BASDAI: Bath Ankylosing Spondylitis Disease Activity Index; CRP: C-reactive protein; ESR: erythrocyte sedimentation rate; TNFi: tumor necrosis factor inhibitor; IL-17i: interleukin-17 inhibitor; DMARD: disease-modifying antirheumatic drug; NSAID: nonsteroidal anti-inflammatory drug.

**Table 2:** Inflammatory markers and disease activity during Upadacitinib treatment (n = 61).

Parameter	Baseline (Month 0)	Month 1	Month 3	*p*-Value
CRP (mg/L)	11.4 ± 10.2	7.8 ± 6.5	6.9 ± 5.8	<0.001
ESR (mm/h)	14.6 ± 11.5	11.2 ± 9.8	10.4 ± 8.7	0.002
BASDAI score	5.8 ± 1.4	4.2 ± 1.3	3.6 ± 1.2	<0.001

Data are presented as mean ± standard deviation. Changes over time were analyzed using repeated measures ANOVA. *p*-values < 0.05 were considered statistically significant.

**Table 3:** Changes in Hematologic, Metabolic, and Muscle Enzyme Parameters During Upadacitinib Treatment (n = 61).

Parameter	Baseline (0 Month)	1 Month	3 Months	*p*-Value
Hematologic				
WBC (×10^3^/μL)	8.85 ± 2.1	8.14 ± 1.8	8.22 ± 1.9	0.064
Hemoglobin (g/dL)	13.6 ± 1.4	13.9 ± 1.2	14.1 ± 1.3	0.032
Lymphocyte (×10^3^/μL)	2.65 ± 0.8	2.42 ± 0.7	2.51 ± 0.8	0.115
Platelet (×10^3^/μL)	315 ± 78	288 ± 65	294 ± 72	0.028
Metabolic & Liver				
ALT (U/L)	24.8 ± 12.4	26.1 ± 14.2	27.5 ± 15.8	0.452
AST (U/L)	21.4 ± 8.6	22.8 ± 9.4	23.1 ± 10.2	0.512
Albumin (g/dL)	4.1 ± 0.4	4.3 ± 0.3	4.4 ± 0.3	0.005
Glucose (mg/dL)	94.6 ± 15.2	96.2 ± 12.8	95.8 ± 14.1	0.841
Creatine Kinase (CK) (U/L)	112.4 ± 54.8	138.6 ± 62.1	144.2 ± 68.5	0.012

Data are presented as mean ± standard deviation. Changes over time were analyzed using repeated measures ANOVA. *p*-values < 0.05 were considered statistically significant.

**Table 4:** Changes in Lipid Parameters During Upadacitinib Treatment (n = 61).

Parameter (mg/dL)	Baseline (0 Month)	1 Month	3 Months	*p*-Value
Total Cholesterol	199.4 ± 41.2	211.8 ± 38.5	215.6 ± 35.4	0.001
LDL-Cholesterol	118.7 ± 33.6	130.2 ± 31.4	136.5 ± 29.8	0.003
HDL-Cholesterol	50.2 ± 12.4	56.4 ± 13.8	58.7 ± 11.5	0.001
Triglycerides	145.8 ± 72.1	154.2 ± 68.4	148.6 ± 62.3	0.412
Non-HDL Cholesterol	149.2 ± 38.5	155.4 ± 36.2	156.9 ± 34.1	0.008
LDL/HDL Ratio	2.36 ± 0.8	2.31 ± 0.7	2.32 ± 0.6	0.845

Data are presented as mean ± standard deviation. Changes over time were analyzed using repeated measures ANOVA. *p*-values < 0.05 were considered statistically significant.

**Table 5:** Changes in disease activity, inflammatory markers, and lipid/albumin parameters from baseline to Month 3 in AS and PsA subgroups.

Variable (Δ Baseline to Month 3)	AS Subgroup (n = 51)	PsA Subgroup (n = 10)	*p*-Value
Δ BASDAI	−2.2 ± 1.1	−2.4 ± 1.3	0.612
Δ CRP (mg/L)	−4.6 ± 3.2	−4.1 ± 3.8	0.658
Δ LDL-C (mg/dL)	+17.4 ± 8.2	+19.8 ± 9.5	0.424
Δ HDL-C (mg/dL)	+8.2 ± 3.9	+10.1 ± 5.2	0.218
Δ LDL/HDL Ratio	−0.04 ± 0.14	−0.06 ± 0.18	0.715
Δ Albumin (g/dL)	+0.32 ± 0.12	+0.28 ± 0.15	0.384

Δ values represent mean ± SD. *p*-values reflect comparisons between AS and PsA subgroups using independent-samples *t*-test or Mann–Whitney U test, as appropriate. BASDAI: Bath Ankylosing Spondylitis Disease Activity Index; CRP: C-reactive protein; LDL-C: low-density lipoprotein cholesterol; HDL-C: high-density lipoprotein cholesterol.

## Data Availability

The original contributions presented in this study are included in the article. Further inquiries can be directed to the corresponding authors.

## Abbreviations

The following abbreviations are used in this manuscript:

AS	Ankylosing Spondylitis
PsA	Psoriatic Arthritis
CVD	Cardiovascular Disease
TNFi	Tumor Necrosis Factor Inhibitor
IL-17i	Interleukin-17 Inhibitor
JAK	Janus Kinase
CRP	C-Reactive Protein
ESR	Erythrocyte Sedimentation Rate
BASDAI	Bath Ankylosing Spondylitis Disease Activity Index
NSAIDs	Non-Steroidal Anti-Inflammatory Drugs
csDMARDs	Conventional Synthetic Disease-Modifying Anti-Rheumatic Drugs
ALT	Alanine Aminotransferase
AST	Aspartate Aminotransferase
CK	Creatine Kinase
TC	Total Cholesterol
LDL-C	Low-Density Lipoprotein Cholesterol
HDL-C	High-Density Lipoprotein Cholesterol
TG	Triglycerides
MACE	Major Adverse Cardiovascular Events
